# Idealized Body Images and Fitness Lifestyles on Social Media: A Systematic Review Exploring the Link Between Social Media Use and Symptoms of Orthorexia Nervosa and Muscle Dysmorphia

**DOI:** 10.1002/erv.70027

**Published:** 2025-08-20

**Authors:** Cristina Vintró‐Alcaraz, Cristina Ballero Reque, Georgios Paslakis, Giulia Testa

**Affiliations:** ^1^ Department of Psychiatry Hospital de Mataró Consorci Sanitari del Maresme (CSDM) Mataró Spain; ^2^ Medical Faculty University Clinic for Psychosomatic Medicine and Psychotherapy Ruhr‐University Bochum (RUB) Luebbecke Germany; ^3^ Instituto de Investigación Transferencia e Innovación Universidad Internacional de La Rioja (UNIR) La Rioja Spain

**Keywords:** bigorexia, muscle dysmorphia, orthorexia nervosa, social media, social networks

## Abstract

**Objective:**

Social media (SM) is a source of appearance‐focused content that promotes idealized bodies and appearances. It also spreads misinformation about nutrition and fitness practices. While SM use has been linked to attitudes toward eating disorders, its association with orthorexia nervosa and muscle dysmorphia—characterized by obsessive behaviors around diet and body image—remains underexplored. This study systematically reviewed quantitative research on the relationship between SM use and symptoms of orthorexia nervosa and muscle dysmorphia.

**Method:**

The review was registered on PROSPERO and adhered to PRISMA guidelines. Twenty‐two studies (orthorexia nervosa: *n* = 17; muscle dysmorphia: *n* = 5) were included, primarily cross‐sectional and conducted on non‐clinical populations. Study quality was assessed using the Quality Assessment Tool for Observational Cohort and Cross‐Sectional Studies.

**Results:**

Most studies found that higher SM use was associated with greater orthorexia nervosa and muscle dysmorphia symptoms, with specific contents –such as posts regarding nutrition, thinness, and exercise– particularly associated with orthorexia nervosa. Platforms like Instagram, Tumblr, and Grindr demonstrated stronger associations with orthorexia nervosa symptoms. Gender differences were also observed.

**Conclusions:**

Findings suggest a link between SM use and orthorexia nervosa symptoms, with preliminary evidence also supporting an association with muscle dysmorphia symptoms. Future research should explore causality and develop targeted prevention and treatment strategies to mitigate these risks.

## Highlights

- Frequent social media use is associated with orthorexia and muscle dysmorphia symptoms.
- Fitspiration and thinspiration content strongly link to orthorexia symptoms.
- Social media addiction shows ties to orthorexia and muscle dysmorphia symptoms.

## 1 Introduction

Social media (SM) use is among the most popular digital activities worldwide, allowing people to share information and communicate with one or multiple users simultaneously (Plackett et al. 2023). It is estimated that in 2023, 4.9 billion people made use of SM, reflecting a global SM use rate of 59%. This figure is expected to rise further, driven by an increasing adoption of mobile devices and enhanced Internet accessibility (Statista 2024). The most widely used platforms today include Facebook, Instagram, Twitter (now X), TikTok, YouTube, Snapchat, Pinterest, Reddit, WhatsApp, and blogs (Kanchan and Gaidhane 2023).

The focus on health and wellness, particularly weight management, is prominently reflected on SM platforms, which have gained popularity over the past decade as informal channels for health information (Kanchan and Gaidhane 2023; Lim et al. 2022; Marks et al. 2020). Content on these platforms often promotes idealized body images, healthy eating, and exercise, attracting considerable attention (Cataldo et al. 2021; de Valle et al. 2021). It is also important to highlight that SM companies employ algorithms that curate and deliver personalized content based on users' past interactions and preferences. While this personalization may enhance user experience, it may also result in biased content selection designed to maximize user engagement and screen time, for example, contributing to focusing attention to unrealistic expectations and thus potentially having adverse effects on body image, particularly among adolescents and young adults (Harriger et al. 2022; Mazzeo et al. 2024).

Recent trends show a proliferation of SM posts, particularly on platforms such as Facebook and Instagram, tagged with ‘thinspiration’, which glorify skinny bodies (Griffiths and Stefanovski 2019). Exposure to such content has been associated with eating disorders (EDs)‐related attitudes and symptoms (González 2023). In addition, there has been an increase in posts tagged ‘fitspiration’, which aim to promote messages about fitness, diet, and appearance (Jerónimo and Carraça 2022). These trends may inadvertently promote unrealistic body ideals, potentially affecting mood, self‐esteem, and emotional well‐being (Cataldo et al. 2021; Griffiths and Stefanovski 2019; Rounds and Stutts 2021).

Previous reviews have pointed to an association between SM use and EDs, suggesting that the content, frequency, or pattern of SM use may be risk factors for EDs‐related psychopathology (Ioannidis et al. 2021; Padín et al. 2021; Wu et al. 2024). However, there is less evidence linking SM use to symptoms of orthorexia nervosa and muscle dysmorphia, two conditions increasingly seen in clinical practice as precursors of disordered eating behaviors or comorbid conditions but not themselves classified as EDs in the latest Diagnostic and Statistical Manual of Mental Disorders 5th edition text revision (DSM‐5‐TR) (American Psychiatric Association 2022). In recent years, increasing criticism has emerged regarding the predominant focus on thinness‐oriented ideals in body image research (McComb and Mills 2022). In contrast, SM often presents content related to healthy eating and fitness as aspirational or educational, potentially normalizing behaviors associated with orthorexia nervosa and muscle dysmorphia. Studying these conditions in relation to SM use expands the field beyond traditional thinness‐driven concerns, allowing for a more contemporary and inclusive understanding of body image issues (Eschrich et al. 2025; Jürgensen et al. 2025).

EDs encompass a range of conditions characterized by significant alterations in eating patterns that imply restriction or overeating/bingeing behaviors. According to the DSM‐5‐TR (American Psychiatric Association 2022) classification, the best‐defined EDs are anorexia nervosa (AN), bulimia nervosa (BN), and binge‐eating disorder (BED). Orthorexia nervosa and muscle dysmorphia (bigorexia) involve an alteration of food intake or body image perception that interferes with daily functioning and may have somatic consequences (Dunn and Bratman 2016; Mosley 2009). However, they are not classified as EDs. While muscle dysmorphia is recognized as a specifier of body dysmorphic disorder, orthorexia nervosa is not included in either the DSM (American Psychiatric Association 2022) or the International Classification of Diseases 11th Revision (ICD‐11) (World Health Organization 2022).

### 1.1 Orthorexia Nervosa

The term orthorexia nervosa was coined by Steven Bratman in 1997, who used the word *orthorexia* (‘orthos’ means *right* or *correct* in Greek, and ‘orexis’ means *appetite*) to describe a condition characterized by an obsessive pattern of eating only food perceived as healthy and ‘pure’ (Bratman and Knight 2000). These restrictive and ritualized eating behaviors may result in health consequences (e.g., nutritional deficiencies) and may also impact the individual's social life (Chaki et al. 2013). This can manifest as avoidance of social gatherings that involve the consumption of foods perceived as unhealthy, or through the potential disapproval of individuals who do not adhere to the same dietary practices (Cena et al. 2019).

Numerous studies indicate that orthorexia nervosa exhibits some overlap with AN and obsessive‐compulsive disorder (OCD) (Atchison and Zickgraf 2022; Duradoni et al. 2023; Pontillo et al. 2022). However, unlike AN and other EDs, orthorexia nervosa places greater emphasis on the quality of foods rather than the quantity consumed. Furthermore, individuals with orthorexia nervosa do not typically experience significant body image distortion or fear of weight gain, which are hallmark features of AN. As for the comparison with OCD, both conditions are marked by rigidity and a tendency to perfectionism; however, the obsessive‐compulsive symptoms associated with orthorexia nervosa are focused only on food (Cena et al. 2019).

While various instruments have been developed to evaluate orthorexia nervosa symptoms (Niedzielski and Kaźmierczak‐Wojtaś 2021; Yargic and Celen 2023), the reported prevalence of this condition exhibits considerable variability. The inconsistencies can be attributed primarily to the lack of consensus regarding the orthorexia nervosa criteria and the suboptimal psychometric properties of some assessment tools (Missbach et al. 2015; Varga et al. 2014). Certain healthcare and sports‐related professions, such as physicians, nurses, and nutritionists demonstrate elevated rates of orthorexia nervosa symptoms in comparison to the general population (Hafstad et al. 2023; Tarı Selçuk and Çevik 2020; Yılmazel 2021), and cultural differences may also play a pivotal role in these findings (Niedzielski and Kaźmierczak‐Wojtaś 2021).

### 1.2 Muscle Dysmorphia/Bigorexia

As previously mentioned, according to the DSM‐5‐TR (American Psychiatric Association 2022), muscle dysmorphia is classified under Body Dysmorphic Disorder. The condition is characterized by a marked dissatisfaction with one's body size and musculature, even in individuals who are objectively muscular, leading to compulsive physical exercise and, at times, the use of anabolic steroids or other substances aimed at increasing muscle mass (Pope et al. 1997). It is associated with considerable distress and deterioration of social functioning. Pope et al. (1993, 1997) first referred to this condition as ‘reverse anorexia’, distinguishing it from AN by highlighting the focus on (allegedly) insufficient muscularity and engagement in muscle‐building behaviors. Later, the presence of obsessive‐compulsive symptoms was emphasized and the similarities between muscle dysmorphia and body dysmorphic disorder were pointed out (Tovt and Kajanová 2021).

There is still ongoing discussion about whether muscle dysmorphia shares more similarities with EDs (Badenes‐Ribera et al. 2019; Murray et al. 2010) or with obsessive‐compulsive spectrum disorders (Cooper et al. 2020). Also, some common characteristics have been noted in relation to addictive disorders (Foster et al. 2015; Olave et al. 2021). It is widely agreed upon that the prevalence of muscle dysmorphia is higher in men than in women, particularly in those who engage in sports focused on increasing muscle mass or gaining strength, such as bodybuilding or weightlifting (Cafri et al. 2008; Olivardia 2001; Olivardia et al. 2000).

### 1.3 Aims

Following all of the above, the present work aimed to systematically review the studies that have evaluated the association between the use of SM and symptoms of either orthorexia nervosa or muscle dysmorphia, to summarize the main findings of the current literature and to identify current gaps and future directions.

## 2 Methods

### 2.1 Search Strategy

This review was conducted according to the PRISMA guidelines (Page et al. 2021) and had been pre‐registered in the PROSPERO database (CRD42024542733). An evidence‐based electronic search was conducted in four databases: PubMed, ProQuest Psychology Database (including: PsycINFO), Web of Science, and Scopus, to which manual searches were added. For each database, a complex search strategy was used, consisting of a combination of Medical Subject Headings (MeSH) terms, keywords, and various terms related to SM use, orthorexia nervosa and muscle dysmorphia. The search terms included: ‘social network*’ OR ‘social media’ OR Instagram OR Facebook OR TikTok OR Twitter OR Pinterest OR Youtube OR you tube OR X OR witter AND orthorexi* OR ortorec* OR ortore* OR ‘obsessive healthy eating’ OR orto‐15 OR orto‐11 OR EHQ OR ‘Eating Habits Questionnaire’ OR ‘muscle dysmorph*’ OR ‘vigorex*’ OR ‘bigorexia’. The software Covidence (Veritas Health Innovation 2024) was used in order to select and organize the studies.

### 2.2 Eligibility Criteria

Search results were limited to original quantitative studies (cross‐sectional, cohort, and case‐control studies) published in English, Spanish, Italian, German, and Greek. All genders at all stages of development were eligible, and no restrictions were placed regarding publication year, given the relatively recent emergence of SM. To be considered, studies had to display quantitative measures of SM use and symptoms of orthorexia nervosa or muscle dysmorphia, including questionnaires, inventories, single questions, scales and subscales.

The eligibility process was carried out in two separate stages: first, two authors independently screened the titles and abstracts of all unduplicated articles and excluded those that were not relevant. The articles selected at this stage then underwent a further review, in which two authors again independently examined the full text for eligibility. In case of disagreement, consensus was reached by a third author at both stages.

Two reviewers independently assessed and extracted the data (e.g., year of publication, study design, sample size and composition, measures of SM use and orthorexia nervosa or muscle dysmorphia symptoms). Again, a third author was consulted as needed to resolve discrepancies.

### 2.3 Quality Assessment

In line with PRISMA guidelines, which recommend evaluating the risk of bias and methodological quality of included studies in systematic reviews, we used the ‘Critical Appraisal Checklist for Analytical Cross‐Sectional Studies’ (Joanna Briggs Institute 2017; Ma et al. 2020). It consists of 14 questions regarding recruitment, sample composition, validity and reliability of the measures used, etc., which were answered independently by two authors (possible categories: ‘Yes’, ‘No’, ‘Cannot Determine’, ‘Not Applicable’, and ‘Not Reported’), resulting in an overall study quality rating of ‘Good’, ‘Fair’, or ‘Poor’.

## 3 Results

### 3.1 Study Selection

**FIGURE 1:**
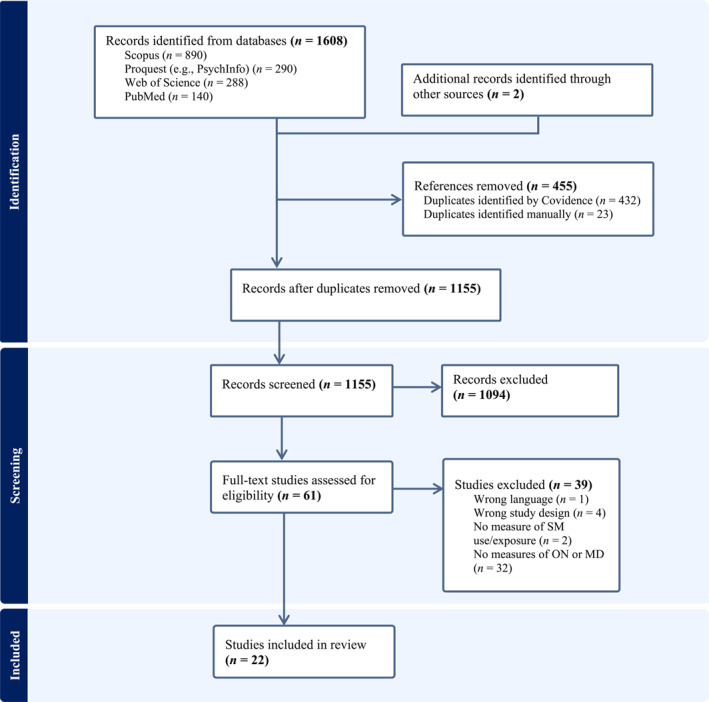
PRISMA flow diagram detailing the selection of articles for the systematic review.

A total of 1608 articles were identified through the database search, with two additional articles retrieved from other sources (refer to Figure 1 for the PRISMA flow diagram). Initially, duplicates were removed using automation tools [Covidence (*n* = 432)] or manually (*n* = 23) by the authors, leaving a total of 1155 articles for title and abstract screening. Overall, 1094 articles were excluded at the title and abstract screening stage, leaving 61 full‐text articles to be assessed for eligibility. Thirty‐nine were then excluded at this stage. Of these, 32 were excluded for lack of quantitative measures of orthorexia nervosa or muscle dysmorphia and the remainders were excluded for the following reasons: language (*n* = 1), study design (*n* = 4) and no measure of SM use (*n* = 2). (See Figure 1).

### 3.2 Overview of the Studies

Twenty‐two studies were included in the systematic review in the end. Seventeen of these studies focused on orthorexia nervosa (Asil et al. 2023; Awad et al. 2024; Christodoulou et al. 2024; Gobin et al. 2021; Hamurcu and Yılmaz 2023; Karniej et al. 2023; Levin et al. 2023; Scheiber et al. 2023; Sener and Ozkaya 2023; Silva et al. 2023; Tarsitano et al. 2022; Turner and Lefevre 2017; Villa et al. 2022; Yılmazel 2021; Yılmazel and Bozdoğan 2020; Yurtdaş‐Depboylu et al. 2022; De Oliveira et al. 2021), while the remaining five studies focused on muscle dysmorphia (Cuadrado et al. 2023; Ganson et al. 2023; Imperatori et al. 2022; Schoenenberg and Martin 2020; Yee et al. 2020). The final sample of participants was *n* = 17,374, divided into studies on orthorexia nervosa (*n* = 13,347) and those on muscle dysmorphia (*n* = 4027).

All articles used a cross‐sectional design, with the exception of one study that collected longitudinal data using ecological momentary assessment (Yee et al. 2020). All were based on non‐clinical samples, except for Sener and Ozkaya (2023) who included overweight and obese individuals recruited from an obesity clinic. Seventeen countries were represented in the included studies, specifically Turkey (*n* = 6); Canada (*n* = 3); Italy (*n* = 2); Brazil (*n* = 2); Germany (*n* = 2); Lebanon (*n* = 1); Greece (*n* = 1); France (*n* = 1); Spain (*n* = 1); Poland (*n* = 1); Portugal (*n* = 1); Austria (*n* = 1); UK (*n* = 1); US (*n* = 1); Chile (*n* = 1); and Australia (*n* = 1). Four of the selected articles included samples from two countries (see Table 1).

**TABLE 1:** Descriptive characteristics of studies included in the systematic review presented in alphabetical order by author name.

First author and year	Country	ON/MD	Sample (% of females)	ON/MD assessments	SM assessment	Key findings
Asil et al. (2023)	Turkey	ON	*N*_total_ = 2526 adult internet users living in Turkey (72% f) Age: M = 28.4 ± 10.3, range *=* 18–65 years	ORTO‐11 (score < 26: Higher orthorexia nervosa tendency)	SMEB Time spent on SM (min/day); content followed on SM	For the participants with orthorexia nervosa tendency (*n* = 1416), the mean age and BMI were significantly higher than for those without orthorexia nervosa tendency (*n* = 1110) No group differences in SMEB Participants with orthorexia nervosa tendency: — Spent less minutes daily on SM than those without orthorexia nervosa tendency — Followed content related to nutrition and physical exercise, and mother‐child education pages more than those without orthorexia nervosa tendency Higher risk of orthorexia nervosa: — In individuals who followed pages with SM content related to nutrition and physical exercise than in those who did not follow these contents — For those not following restaurant and entertainment pages
Awad et al. (2024)	Lebanon	ON	*N*_total_ = 363 university students (61.7% f) Age: M = 22.65 ± 3.48 years	TOS	SMD Frequency of use of SM (number of times of entry SM, time spent on SM)	SMD was positively associated with orthorexia nervosa symptoms, and this association was mediated by loneliness Orthorexia nervosa symptoms were positively associated with physical activity index More orthorexia nervosa symptoms were significantly associated with: — Using Instagram or Tumblr — Spending 30–60 min per day on SM compared to less than 30 min — Higher physical activity index — Loneliness
Christodoulou et al. (2024)	Greece	ON	*N*_total_ = 407 adults (68.3% f) Age: range = 18–65 years	ORTO‐R	Two questions on Instagram use (presence of an active Instagram account; average daily time dedicated to Instagram)	Women compared to men had statistically significant higher scores of ORTO‐R Significant difference in the means of ORTO‐R scores for different daily use of Instagram One‐unit increment in the ORTO‐R score was associated with a 10% elevation in the odds of participants spending > 3 h/day on Instagram in comparison to individuals spending < 1 h/day on the platform Women exhibited nearly three times higher odds of engaging in Instagram usage for > 3 h/day, after a one‐point increase in the ORTO‐R score
Cuadrado et al. (2023)	France	MD	*N*_total_ = 342 students practicing weightlifting at the university gym (23.7% f) Age: M = 20.95 ± 2.99, range = 17–32 years	MDDI	Frequency and type of SM pro‐muscularity used Muscle pics use of SM for taking and sharing photos of body muscularity	18.7% of the students (*n* = 64) showed muscle dysmorphia symptomatology Muscle pics were linked to the total MDDI score, and to its subscales ‘drive for size’ and ‘functional impairment’ (but not to the ‘appearance intolerance’ subscale) Use of pro‐muscularity SM was correlated to ‘muscle pics’ and muscle dysmorphia Among individuals with muscle dysmorphia, risk factors identified included higher use of pro‐muscularity websites and muscle pics
De Oliveira et al. (2021)	Brazil	ON	*N*_total_ = 285 undergraduate students in nutrition (91.6% f) Age: range = 18–23 years	ORTO‐15 (score < 40: Risky for orthorexia nervosa)	Frequency and use of SM	72% (*n* = 206) of the participants showed risk for orthorexia nervosa Higher prevalence in the frequency and use of SN among the students at risk of orthorexia nervosa compared to the students without risk
Ganson et al. (2023)	Canada	MD	*N*_total_ = 2538 adolescents and young adults resident in Canada (58.2% f) Age: M_female_ = 23.1 ± 3.9, M_male_ = 22.8 ± 3.9, range = 16–30 years	MDDI	Daily frequency of visiting SM (i.e., Facebook, Twitter, Instagram) and video chatting (i.e., Skype, Facetime)	Women reported significantly greater time on SM compared to men Significant associations between screen time and symptoms of muscle dysmorphia (MDDI score) SM use was most strongly associated with symptoms of muscle dysmorphia among men, whereas video chatting was most strongly associated with symptoms of muscle dysmorphia among women
Gobin et al. (2021)	Canada	ON	*N*_total_ = 143 (100% f) Age: M = 25.85 ± 8.1, range = 17–32 years	EHQ (median split of the total score to define low/high ON symptoms)	Self‐reported increases or decreases in SM habits since the COVID‐19 lockdown began	After the lockdown, women with high orthorexia nervosa symptoms, compared to those with low orthorexia nervosa symptoms, reported: — Eating a lot more than usual — Feeling greater pressure to diet and lose weight — Thinking about food more often than usual — Experiencing greater weight gain — Perceiving more pressure from SM, specifically to lose weight and to exercise Women with high EHQ ‐problems reported seeing more weight loss content in their SM compared to those with low scores Those with low EHQ‐Feelings reported feeling less pressure to lose weight or exercise from SM compared to those with higher scores
Hamurcu and Yılmaz (2023)	Turkey	ON	*N*_*t*otal_ = 339 nursing students using at least one SM (84.4% f) Age: M = 20.74 ± 1.93, range *=* 18–37 years	ORTO‐11 (score ≤ 25: Higher orthorexia nervosa tendency)	Sociodemographic form including SM usage and characteristics (type of SM tool, frequency of use, influence of SM on individual's own decisions) SMUIS	30.38% (*n* = 103) of the students showed higher orthorexia nervosa tendency versus 69.62% (*n* = 236) that showed lower orthorexia nervosa tendency Orthorexia nervosa tendency was found to increase accordingly with the duration of SM use and the influence of SM on their own decisions Orthorexia nervosa tendency did not differ according to the type of SM tools used Negative association between SMUIS (total score and the ‘integration into social routines’ sub‐dimension) and the ORTO‐11 scores, suggesting higher orthorexia nervosa tendency for higher SMUIS
Imperatori et al. (2022)	Italy	MD	*N*_total_ = 721 participants, (69.9% f). Age: M = 24.13 ± 3.70, range = 18–24 years	MDDI (score > 39: Clinically relevant muscle dysmorphia)	BSMAS (score ≥ 19: problematic SM use)	BSMAS was significantly associated with both muscle dysmorphia and ED‐related symptoms The relationship between BSMAS and eating disorders was mediated by the severity of muscle dysmorphia‐related symptoms, controlling for relevant confounding factors (e.g. sex, age, educational level, marital status, job status, BMI, problematic alcohol use, tobacco use, illicit drug use, psychopathological distress)
Karniej et al. (2023)	Spain and Poland	ON	*N*_total_ = 394 male (0% f) with homosexual orientation (*n* = 188 from Poland; *n* = 206 from Spain) Age: M_Poland_ = 32 ± 8.58 years; M_Spain_ = 43 ± 10.56 years	ORTO‐15 (score < 35: Risk for orthorexia nervosa)	Self‐administered questionnaire on use of SM (Facebook, Instagram, Twitter, others) and dating app (Grindr)	33% of the total sample showed risk of orthorexia nervosa The Polish group had a higher risk for orthorexia nervosa (40% *vs*. 8%) and used the Grindr dating app more often (65% *vs*. 35%) than the Spanish group The use of Instagram was associated with a 52.1% lower risk of orthorexia nervosa, while the use of the Grindr application increased this risk more than 5 times The most important predictors of orthorexia nervosa were low BMI and the use of Grindr. Grindr use was associated with higher risk of orthorexia nervosa
Levin et al. (2023)	Canada	ON	*N*_total_ = 333 University students (72% f) Age: M = 20.9 ± 4.3	EHQ	Number of hours/day of ‘view healthy eating content’/‘share healthy eating content’	Cluster analysis showed 3 clusters associated with orthorexia nervosa: — Orthorexia nervosa subtype 1 (orthorexia nervosa/ED combined) — Orthorexia nervosa subtype 2 (orthorexia nervosa/ED combined, without weight/shape concerns) — Orthorexia nervosa subtype 3 (orthorexia nervosa only) Other clusters were: low psychopathology; poor body image; primarily ED; normative group (reference group) Being female was associated with being in the primarily ED group, whereas those in orthorexia nervosa subtype 3 were more likely to be male The orthorexia nervosa/ED (subtype 1 and 2) and the primarily ED groups were both associated with higher BMI In terms of SM use, being in the orthorexia nervosa subtype 2 was associated with spending more time both viewing and sharing content related to healthy eating on SM
Scheiber et al. (2023)	Germany and Austria	ON	*N*_total_ = 647 (54.6% female). Age: M = 23.7 ± 4.7, range = 18–30 years	DOS	Involvement with health and fitness accounts on SM (3 questions) Appearance comparison in SM (6 questions)	Involvement with interest in health and fitness accounts on SM was positively related to orthorexia nervosa tendencies, and this relation was mediated by body image variables (thin‐ and muscularity ideal internalizations) More significant involvement with health and fitness accounts on SM influenced higher appearance comparisons. However, appearance comparisons (upward comparisons) were unrelated to the orthorexia nervosa tendencies
Schoenenberg and Martin (2020)	Germany	MD	*N*_total_ = 203 males (0% f) exercising regularly and using Instagram. Age: M = 27.98 ± 7.5 years	— DMS — MDDI — Self‐screening for body dysmorphic disorder	Time spent on SN use (time per day) Time spent on #fitspiration pictures on Instagram	Positive association between use of Instagram and DMS and between the use of fitspiration pictures and DMS Exclusive weightlifters showed a significantly higher drive for muscularity than strength and endurance athletes In a comparison of strength athletes with power and ball sports athletes, the former showed significantly higher use of fitspiration images, drive for muscularity, and expression of muscle dysmorphia symptoms The drive for muscularity and the severity of muscle dysmorphia symptoms was significantly predicted by the frequency of Instagram use, and this relationship was mediated by the internalization of the media ideal of beauty The frequency of use of fitspiration pictures significantly predicted the pursuit of muscularity and the severity of muscle dysmorphic symptoms Partial mediation by the tendency to make appearance‐related comparisons was found in the case of striving for muscularity
Sener and Ozkaya (2023)	Turkey	ON	*N*_total_ = 174 overweight/obese outpatients of an obesity clinic (67.2% f). Age: range = 18–65 years	ORTO‐11	SMBÖ‐YF	There were no differences in mean ORTO‐11 score between overweight and obese patients. Negative association between the ORTO‐11 score and the virtual communication sub‐dimension of the SMBÖ‐YF, suggesting that higher orthorexia nervosa tendency was associated with higher SM addiction in the sample
Silva et al. (2023)	Portugal and Brazil	ON	*N*_total_ = 238 Instagram users (64% f) from Portugal (*n* = 138), and Brazil (*n* = 100) Age: range Portugal = 25–50 years; range Brazil = 18–35 years	ORTO‐15	Instagram use scale Exposure to fitness content on Instagram (fitspiration)	Instagram use and exposure to fitspiration were not directly related to orthorexia nervosa symptoms and body dissatisfaction However, Instagram use and exposure to fitspiration led to increased internalization of the athletic ideal and social comparisons, which in turn were associated with increased body dissatisfaction and, as a result, increased orthorexia nervosa symptoms Perfectionism was a risk factor for the development of body dissatisfaction and orthorexia nervosa Similar results in the Portuguese and Brazilian participants, suggesting no significant role of cultural differences
Tarsitano et al. (2022)	Italy	ON	*N*_total_ = 4107 adults (95.4% f). Age: *M* = 31 ± 9 years	I‐DOS (score ≥ 35: ON risk)	Likert scales to assess type of SM used, time spent on SM; time spent on SM with content related to food/physical activity/weight loss	The risk of orthorexia nervosa was present in 28.5% of the sample Participants were mostly Instagram users. 52% of the Instagram users reported seeing photos of food, and 38.5% of physical activities. 50.2% of the Instagram users reported that they rarely searched for photos of dietary or weight loss products The prevalence of orthorexia nervosa was higher among participants who reported a SM use of > 60 min (31%) as compared to those reported to use SM for < 15 min/day (21.8%) Positive association between I‐DOS score average time spent on SM channel per day
Turner and Lefevre (2017)	44.6% UK, 26.7% US, (remaining from 40 other countries)	ON	*N*_total_ = 680 (100% f). Age: *M* = 24.70 ± 7.87, range = 18–75 years	ORTO‐15 (two cut‐off‐scores for orthorexia nervosa. Tendency: < 40 and < 35)	Number of SM (choosing among: Instagram, Facebook, Twitter, Pinterest, Google+, Tumblr, and LinkedIn) Frequency of use of SM	All but one participant used at least one SM, Instagram being the most popular (95% daily users) 80% of the Instagram users ranked food as the 1st or 2nd most frequent image category appearing on their Instagram feed The prevalence of orthorexia nervosa among Instagram users (*n* = 669) was 90.4% (< 40 cut‐off) or 49.3% (< 35 cut‐off) Negative association between ORTO‐15 score and frequency of Instagram use, with higher Instagram use being associated with a greater tendency towards orthorexia nervosa No relationship between ORTO‐15 scores and SM channels other than Instagram (with the exception of Twitter, which showed a small positive correlation) Significant predictors of ORTO‐15 in the regression included country of residence (UK/non‐UK) and Instagram use
Villa et al. (2022)	Chile	ON	*N*_total_ = 90 students of nutrition and dietetic (87.8% f) Age: M = 22.2 ± 2.6 years	ORTO‐11‐ES (score < 25: Risk for orthorexia nervosa)	Daily Instagram use (minutes), provided by the app settings and then classified in three categories (< 1 h, 1–3 h, and> 3 h)	Orthorexia nervosa risk prevalence in the sample was 23.3% Time spent on the social network Instagram (< 1 h OR 2.77, > 3 h OR 1.80) was associated with increased risk for orthorexia nervosa Only for the population at risk for orthorexia nervosa, the number of minutes used on Instagram were positively associated with the ORTO‐11‐ES score
Yee et al. (2020)	Australia	MD	*N*_total_ = 223 men (0% f) holding an iPhone Age: M = 20.89 ± 4.39, range = 18–52 years	MBAS‐R (subscales: ‘Body fat’, ‘muscularity’) Likert scale on: — State body fat — Muscularity dissatisfaction — Urge to engage in body change behavior	View of fitspiration and view of thinspiration contents	Viewing fitspiration compared to neutral images increased state body fat dissatisfaction, muscularity dissatisfaction, negative mood, and the urge to reduce body fat and increase muscularity Viewing thinspiration relative to neutral images reduced both state body fat and muscularity dissatisfaction Trait muscularity dissatisfaction moderated the impact of viewing fitspiration and thinspiration images on state muscularity dissatisfaction Trait body fat dissatisfaction moderated the effects of thinspiration but not fitspiration exposure on state muscle dissatisfaction Trait appearance comparison moderated the effects of exposure to fitspiration imagery on both state body fat and muscularity dissatisfaction
Yılmazel and Bozdoğan (2020)	Turkey	ON	*N*_total_ = 969 medical and nursing students using SN (63.9% f). Age: M = 21.4 ± 3.2 years	ORTO‐15 (score < 40: Orthorexia nervosa tendency)	SM use (yes/no) SMAS.	SM addiction in 78.8% of participants (higher prevalence in nurses than doctors); and 62.2% of participants had orthorexia nervosa tendencies (similarly distributed across age and genders) 31.0% of high/very high SM addicts had an orthorexia nervosa tendency. Orthorexia nervosa tendency was 1.37 times higher in SM addicts
Yılmazel and Bozdoğan (2020)	Turkey	ON	*N*_total_ = 420 school teachers (46.2% f). Age: M = 43.4 ± 7.5, range = 18–51 years	ORTO‐15 (score < 40: Orthorexia nervosa tendency)	Type of SN used (between Instagram, Facebook, Twitter, Pinterest, Google+, Youtube, Snapchat and LinkedIn)	Female gender, Instagram use, and limited health literacy were significantly associated with higher orthorexia nervosa tendency. Limited health literacy increased the risk of orthorexia nervosa 4.85 times
Yurtdaş‐Depboylu et al. (2022)	Turkey	ON	*N*_*t*otal_ = 1232 high school adolescents (57.8% f) Age: *M* = 15.7 ± 1.19, range = 13–18 years	ORTO‐11 (≤ 27 scores for orthorexia nervosa tendency)	SMAS‐A	27.9% (*n* = 344) were at risk of orthorexia nervosa *vs*. 72.1% of students (*n* = 888) without orthorexia nervosa tendency SM addiction level of 4.4% of adolescents was high, 33.5% was moderate, and 62.1% was low. Higher SM addiction levels in girls than boys SM addiction was significantly associated with a greater risk of ED, lower body image, and orthorexia nervosa tendency Adolescents who stated that they always or often read nutrition‐related posts on SM had an increased likelihood of ED risk and orthorexia nervosa tendencies

Abbreviations: BMI, body mass index; BSMAS, Bergen Social Media Addiction Scale; DMS, Drive for Muscularity Scale; DOS, Düsseldorf Orthorexia Scale; ED, eating disorders; EHQ, Eating Habits Questionnaire; f, females; I‐DOS, Italian‐Düsseldorf Orthorexia Scale questionnaire; M, mean; MBAS‐R, muscularity subscale of the revised male body attitudes scale; MD, muscle dysmorphia; MDDI, muscle dysmorphic disorder Inventory; ON, orthorexia nervosa; SM, social media; SMAS (SMAS‐A), Social Media Addiction Scale (A: adolescents); SMBÖ‐YF, Social Media Addiction Adult Form Scale; SMD, Social Media Disorder Scale; SMEB, Social Media and Eating Behavior Scale; SMUIS, Social Media Use Integration Scale; TOS, Teruel Orthorexia Scale.

In terms of gender, two studies included only women (Gobin et al. 2021; Turner and Lefevre 2017), while three focused exclusively on men (Karniej et al. 2023; Schoenenberg and Martin 2020; Yee et al. 2020), with Karniej et al. (2023) only including homosexual men. The remaining 17 studies included mixed gender samples, although higher proportions of women were usually reported, particularly in those studies focusing on orthorexia nervosa.

In terms of age, the majority of studies were conducted with adults, while three studies recruited youths at the age of 16–17 (Cuadrado et al. 2023; Ganson et al. 2023; Gobin et al. 2021), and only one focused on school‐aged adolescents (Yurtdaş‐Depboylu et al. 2022). Although not all studies specified the sample's age range, the overall sample was (estimated to be) between 13 and 75 years old. Notably, seven studies included university students, particularly from the healthcare fields (Awad et al. 2024; Cuadrado et al. 2023; De Oliveira et al. 2021; Hamurcu and Yılmaz 2023; Levin et al. 2023; Villa et al. 2022; Yılmazel 2021), and one study focused on school teachers (Yılmazel and Bozdoğan 2020).

#### 3.2.1 Assessment of Orthorexia Nervosa and Muscle Dysmorphia

The assessment of orthorexia nervosa was carried out using a heterogeneous set of self‐report measures. Twelve studies used the ORTO‐15 or ORTO‐11 scales (Asil et al. 2023; Christodoulou et al. 2024; De Oliveira et al. 2021; Hamurcu and Yılmaz 2023; Karniej et al. 2023; Silva et al. 2023; Turner and Lefevre 2017; Villa et al. 2022; Yılmazel 2021; Yılmazel and Bozdoğan 2020; Yurtdaş‐Depboylu et al. 2022), which are the first questionnaires developed to assess orthorexia nervosa tendencies. Two studies (Gobin et al. 2021; Scheiber et al. 2023) used the more recent Düsseldorf Orthorexia Scale (DOS) (Barthels et al. 2015), while two studies (Gobin et al. 2021; Levin et al. 2023) used the Eating Habits Questionnaire (EHQ) (Gleaves et al. 2013). Only the study from Awad et al. (2024) used the Teruel Orthorexia Scale (TOS), a multidimensional measure of both pathological and non‐pathological orthorexia (Barrada and Roncero 2018).

Regarding muscle dysmorphia assessments, four studies (Cuadrado et al. 2023; Ganson et al. 2023; Imperatori et al. 2022; Schoenenberg and Martin 2020) used the Muscle Dysmorphic Disorder Inventory (MDDI), evaluating three dimensions: drive for size, appearance intolerance, and functional impairment (Hildebrandt et al. 2004). Only one of these studies established a cut‐off point (> 39) to indicate the clinical relevance of muscle dysmorphia symptoms (Imperatori et al. 2022). Some participants were also evaluated using the Drive for Muscularity Scale (DMS), which measures one's desire for muscularity (McCreary 2007), while others were evaluated using some items from the Revised Male Body Attitudes Scale (MBAS‐R) as well as questions related to state body fat and muscularity dissatisfaction (Ryan et al. 2011).

### 3.3 Assessment of SM Use and Addiction

Most of the reviewed studies used ad hoc items or scales to evaluate SM use, with frequency, type of SM, and content related to nutrition and physical exercise (e.g., dieting, fitspiration) being the main features addressed. All the measures were self‐reported, except for those of Villa et al. (2022), who assessed daily Instagram use in minutes through smartphone app settings. Asil et al. (2023) used the Social Media and Eating Behavior Scale (SMEB), which is a standardized questionnaire to assess the association between SM use and specific eating behaviors (Keser et al. 2020).

Four studies (Imperatori et al. 2022; Sener and Ozkaya 2023; Yılmazel 2021; Yurtdaş‐Depboylu et al. 2022) also assessed problematic or addictive use of SM using standardized instruments, including the following: the Bergen Social Media Addiction Scale (BSMAS) (Schou Andreassen et al. 2016); the Social Media Addiction Adult Form Scale (SMBÖ‐YF) (Şahin and Yağcı 2017); the Social Media Disorder Scale (SMD) (van den Eijnden et al. 2016); and the Social Media Addiction Scale for adolescents or adults (Tutgun‐ünal and Deniz 2015).

### 3.4 Relationship Between SM and Orthorexia Nervosa

The frequency of use and time spent on SM were assessed by seven studies out of 17, all of which found significant associations with symptoms of orthorexia nervosa. Regarding the direction of the association, five studies reported positive associations between frequency of use or time spent on SM and symptoms of orthorexia nervosa in university students or adults from the general population (Awad et al. 2024; Christodoulou et al. 2024; De Oliveira et al. 2021; Hamurcu and Yılmaz 2023; Tarsitano et al. 2022). Villa et al. (2022) found mixed results; both low Instagram use (under 1 h/day) and very high use (over 3 h/day) were linked to a higher risk of orthorexia nervosa in nutrition and medical students. However, among those already at higher risk, more time spent on Instagram, measured continuously, was clearly associated with higher orthorexia scores (Villa et al. 2022). Finally, one study reported that adults with orthorexia nervosa tendencies spent fewer minutes per day on SM than those without orthorexia nervosa tendencies (Asil et al. 2023).

Regarding the type of SM platform, Instagram use was associated with higher orthorexia nervosa tendencies in school teachers (primarily women) (Yılmazel and Bozdoğan 2020), university students (Awad et al. 2024), and adult women in general (Turner and Lefevre 2017). Awad et al. (2024) also identified an association between Tumblr use and increased orthorexia nervosa risk, while Turner and Lefevre (2017) reported a small protective effect of Twitter against orthorexia nervosa risk. A study of homosexual men found that Instagram use was linked to a lower risk of orthorexia nervosa compared to Grindr (gay dating app) use, with Grindr use increasing orthorexia nervosa risk more than fivefold (Karniej et al. 2023). Hamurcu and Yılmaz (2023) found no significant relationship between the type of SM platform used and orthorexia nervosa risk among nursing students.

Eight studies examined specific SM content. Turner and Lefevre (2017) found that food was one of the most frequent image categories in participants' Instagram feeds, and Tarsitano et al. (2022) reported that 52% of Instagram users viewed food‐related photos, while 38.5% viewed physical activity content. However, 50.2% of users rarely searched for photos related to diets or weight loss products (Tarsitano et al. 2022). Meanwhile, others reported associations between orthorexia nervosa tendencies and viewing SM content related to healthy eating and exercise/fitness (Asil et al. 2023; Gobin et al. 2021; Levin et al. 2023; Scheiber et al. 2023). Silva et al. (2023) found that exposure to fitspiration content on Instagram facilitated the internalization of the athletic ideal, promoting both upward and downward appearance comparisons, with individuals comparing themselves to those they perceive as more (upward) or less (downward) attractive. These factors, in turn, were associated with greater body dissatisfaction and increased symptoms of orthorexia nervosa. Scheiber et al. (2023) also found that body image variables (e.g., thinness ideal and muscle ideal internalization) mediated the relationship between orthorexia nervosa and healthy eating behaviors. Scheiber et al. (2023) did not find a direct relationship between appearance comparisons and orthorexia nervosa tendencies, although greater involvement in health and fitness accounts on SM was associated with more frequent appearance comparisons. In the only study on adolescents, Yurtdaş‐Depboylu et al. (2022) found that the likelihood of orthorexia nervosa and ED tendencies was higher among those who frequently read nutrition‐related posts on SM. Regardless of the specific content viewed, individuals with orthorexia nervosa tendencies reported feeling higher pressure from SM to lose weight and exercise (Gobin et al. 2021), and stated that SM influenced their personal decisions (Hamurcu and Yılmaz 2023).

Four studies examined the relationship between problematic or addictive SM use and orthorexia nervosa. In a sample of overweight/obese outpatients from an obesity clinic, higher orthorexia nervosa tendencies were associated with higher SM addiction scores (Sener and Ozkaya 2023). Among university students, Yılmazel (2021) observed that those classified as SM addicts (78.8% of participants) had higher orthorexia nervosa tendencies. Awad et al. (2024) found that symptoms of SM use disorder were positively associated with orthorexia nervosa symptoms, with loneliness mediating this association. Finally, among adolescents, SM addiction was linked to greater orthorexia nervosa tendencies, increased risk of EDs, and lower body image satisfaction (Yurtdaş‐Depboylu et al. 2022).

### 3.5 Relationship Between SM and Muscle Dysmorphia

The five studies that focused on muscle dysmorphia showed associations between muscle dysmorphia symptoms and the use of SM. Some authors highlighted the link between muscle dysmorphia pathology and exposure to fitness‐related contents on social networks. For example, three studies found that fitspiration and muscle‐related content were associated with muscularity dissatisfaction, negative mood when interrupting exercise routines, a tendency to make appearance‐based comparisons with others, and the internalization of beauty ideals (Cuadrado et al. 2023; Schoenenberg and Martin 2020; Yee et al. 2020). Another study found that fitspiration images increased men's body dissatisfaction, whereas thinspiration images decreased body dissatisfaction; viewing either fit‐ or thinspiration images was associated with higher urges to increase muscularity (Yee et al. 2020). In the same study, men with greater baseline dissatisfaction with their muscularity were most prone to muscularity dissatisfaction following exposure to fitspiration images (Yee et al. 2020).

On the other hand, Ganson et al. (2023) did not focus on the content, but rather on the time spent and the platforms themselves. The authors found that among women ‐who overall spent more time on social networks than men‐video chatting (i.e., FaceTime, Skype) was associated with muscle dysmorphia symptoms. In men, however, these symptoms were more closely related to common social networks like Instagram, Facebook, and Twitter.

The study by Imperatori et al. (2022) showed a significant association between problematic SM use and both muscle dysmorphia and ED symptomatology. In fact, muscle dysmorphia‐related symptoms mediated the association between problematic SM use and ED.

### 3.6 Quality Assessment

Of the 17 studies focusing on orthorexia nervosa, most provided a clear objective and description of the study population. All studies used reliable and valid outcome measures. However, the lack of statistical control for confounding variables and the predominance of cross‐sectional designs led to a lower overall quality rating in many cases.

In the five muscle dysmorphia studies, the research questions were well defined, and all but one study examined different levels of SM exposure and used reliable outcome measures, although the quality of the exposure measures was not always clearly reported.

The overall quality of the included studies was heterogeneous, with ratings ranging from ‘fair’ (*n* = 11) to ‘good’ (*n* = 8). Three studies were rated as ‘poor’, mainly due to limited sample descriptions, lack of justification for sample size, unclear validity of the questionnaires used, and insufficient control of confounding variables. Although no studies were excluded based on quality, these assessments were used to contextualize and qualify the interpretation of the findings, particularly when studies yielded inconsistent results. Quality ratings for each study are presented as Table S1.

## 4 Discussion

This systematic review investigated the relationship between SM use and symptoms of orthorexia nervosa or muscle dysmorphia. Most studies included in the review focused on orthorexia nervosa, while a limited number examined individuals with symptoms of muscle dysmorphia. Various SM‐related factors were analyzed in terms of their association with these conditions. In terms of population, a higher prevalence of women was found to be at risk for orthorexia nervosa, while men were more commonly at risk for muscle dysmorphia. This likely reflects gender‐specific risk factors associated with each condition (Grossbard et al. 2011; Łucka et al. 2024).

First, higher frequency and more time spent on SM in general were associated with elevated symptoms of orthorexia nervosa and muscle dysmorphia in nearly all studies assessing these factors (Awad et al. 2024; De Oliveira et al. 2021; Ganson et al. 2023; Hamurcu and Yılmaz 2023; Tarsitano et al. 2022; Turner and Lefevre 2017; Villa et al. 2022). This is consistent with previous research showing associations between general SM use, particularly image‐focused platforms, and symptoms related to EDs (Dane and Bhatia 2023; Padín et al. 2021). Several of these studies were rated as high quality (e.g., Awad et al. 2024; Tarsitano et al. 2022; Villa et al. 2022), lending additional weight to their findings. Despite only seven out of the 17 studies on orthorexia nervosa evaluating the frequency of use or time spent on SM, the findings from higher‐quality research support a prudent, but meaningful interpretation of potential inferences.

Only one study in this review highlighted potential discrepancies, showing that time spent on SM was not associated with orthorexia nervosa risk, whereas following SM content on nutrition and physical exercise was (Asil et al. 2023). This underscores the relevance of the specific content viewed or shared in SM, such as the use of fitspiration or thinspiration content, which has been associated directly with orthorexia nervosa tendencies (Asil et al. 2023; Gobin et al. 2021; Levin et al. 2023; Scheiber et al. 2023; Yurtdaş‐Depboylu et al. 2022) or indirectly, through internalization of the athletic ideal and social comparisons (Silva et al. 2023). Although fewer studies have been conducted on muscle dysmorphia symptoms, preliminary evidence suggests an association between fitspiration content on SM and muscle dysmorphia, whereas thinspiration has been less explored (Cuadrado et al. 2023; Schoenenberg and Martin 2020; Yee et al. 2020).

Some studies examined the type of SM, suggesting that Instagram, Tumblr and Grindr are associated with orthorexia nervosa tendencies. However, the large heterogeneity of SM platforms assessed across studies (e.g., some studies focused only on Instagram without considering other SMs), complicates cross‐platform comparisons, thereby hindering firm conclusions based on the current data. Furthermore, the constant emergence of new SM platforms and trends requires constant updates of assessments. Nevertheless, special attention should be directed to those platforms that allow the sharing or viewing of image or video‐based content, which is expected to have a greater impact, as shown in the case of ED‐related psychopathology (Padín et al. 2021).

The findings of this review are overall consistent with ED literature, linking fitspiration/thinspiration content to the development of ED‐related attitudes and symptoms (González 2023; Griffiths and Stefanovski 2019; Jerónimo and Carraça 2022). Recently, Dane and Bhatia (2023) proposed that certain SM exposures and individual risk factors can influence (strengthen) the relationship between SM and ED. Interpreting our findings within this framework, specific features of SM (e.g., engagement with fitspiration and thinspiration trends) might contribute to body image concerns and orthorexia nervosa and muscle dysmorphia symptoms, and this relationship may be mediated by other factors such as thin/fit ideal internalization and appearance comparisons. Furthermore, other variables related to body image, such as body or muscularity dissatisfaction and internalization of thinness or muscularity ideals were also identified as potential mediators of the relationship between exposure to specific SM content and symptoms of orthorexia nervosa (Silva et al. 2023) or muscle dysmorphia (Cuadrado et al. 2023; Schoenenberg and Martin 2020; Yee et al. 2020). These effects are further exacerbated by algorithms used by SM platforms, which expose users to personalized content aimed at maximizing engagement, often at the expense of their well‐being (Harriger et al. 2022). As such, SM companies bear significant responsibility, and regulatory efforts have begun to address this issue (e.g., The Digital Services Act, European Commission 2022).

Problematic or addictive SM use is another risky behavior that has been associated with higher orthorexia nervosa tendencies across various sample groups, including individuals with obesity, university students, and adolescents (Awad et al. 2024; Sener and Ozkaya 2023; Yılmazel 2021; Yurtdaş‐Depboylu et al. 2022). A single study was identified showing an association between problematic SM use and muscle dysmorphia symptoms, with muscle dysmorphia acting as a mediator between problematic SM use and ED symptomatology (Imperatori et al. 2022). The high quality of this study supports the relevance of this relationship. These findings expand on previous research linking ED psychopathology with problematic Internet use, which includes, but is not limited to, SM addiction (Hinojo‐Lucena et al. 2019; Ioannidis et al. 2021). Currently, SM addiction, like other proposed behavioral addictions, is not recognized by the DSM‐5 or ICD‐11, although common assessment criteria include frequency/engagement, difficulty controlling the behavior, mood regulation through SM use, and symptoms of tolerance and withdrawal.

Growing concerns about the impact of SM addiction on mental health (Blanchard et al. 2023; Szczygieł and Podwalski 2020) are supported by this review. It confirms a link between SM addiction and orthorexia nervosa tendencies, with preliminary evidence also pointing to an association with muscle dysmorphia. One potential explanation is that individuals with SM addiction likely spend a considerable amount of time on these platforms, where frequent exposure to thinspiration or fitspiration content may heighten their risk of developing orthorexia nervosa or muscle dysmorphia. Alternatively, individuals already at risk of orthorexia nervosa or muscle dysmorphia may be drawn to SM content focused on food and fitness, thereby increasing their vulnerability to SM addiction.

Several limitations of the current review must be acknowledged. First, the included studies mainly used cross‐sectional design, which prevents establishing causality between variables, underscoring the need for longitudinal research to investigate risk factors. In fact, only one study employed ecological momentary assessment at multiple time points to capture temporal changes in muscle dysmorphia (Yee et al. 2020). Another important limitation is the high heterogeneity of the studies included, which prevented us from conducting a meta‐analysis. Specifically, the variables for SM use vary significantly, not only in terms of assessment instruments, but also in the constructs related to SM use (e.g., frequency of use and time spent, SM platform, content of SM, symptoms of SM addiction). Additional sources of heterogeneity stem from the conditions studied (i.e., orthorexia nervosa or muscle dysmorphia) and the differences in the type of analysis conducted (correlational vs. group comparisons). Therefore, we consider a systematic review with a narrative synthesis to be the most suitable approach for providing a comprehensive overview of the current literature in this field. Another limitation concerns the self‐reported nature of the assessment measures used across studies. This is particularly relevant for SM use, where potential biases in reporting time spent or use patterns may arise. Future research should aim to incorporate more objective measures, such as in the study by Villa et al. (2022), which tracked daily Instagram use in minutes through app settings. Finally, while most studies focused on young adults (e.g., university students), research on adolescents is notably lacking (with only one study identified), even though adolescents are considered a high‐risk group for the effects of SM exposure and should be studied longitudinally. The same applies to the investigation of sexual and gender minorities; studies in these cohorts are missing, but urgently needed to assess specific risk factors and needs.

Despite these limitations, this review is the first to systematically examine the link between SM use and orthorexia nervosa and muscle dysmorphia tendencies. While previous research has explored the connection between SM and ED psychopathology, it is important to recognize that orthorexia nervosa and muscle dysmorphia are emerging clinical or subclinical conditions distinct from ED, although they share overlapping symptoms. Although the current evidence base is mixed, the associations found in higher‐quality studies suggest a meaningful potential link between SM use and these conditions ‐particularly in relation to problematic use, exposure to specific content (e.g., fitspiration), and internalization of body ideals. The pervasive messages about healthy lifestyles and healthy eating on SM may be particularly harmful to susceptible individuals and could increase the risk of orthorexia nervosa or muscle dysmorphia symptoms.

These findings suggest the need to adapt existing prevention and treatment programs to mitigate risks associated with SM exposure, particularly regarding fitspiration and thinspiration contents. Such adaptations should address both universal prevention at early ages as well as targeted secondary prevention for at‐risk populations.

## 5 Conclusions

Higher SM use, particularly exposure to fitspiration and thinspiration content, is linked to higher symptoms of orthorexia nervosa and, as preliminary evidence indicates, muscle dysmorphia symptoms. Body image‐related factors and appearance concerns emerge as key moderators in these relationships. Additionally, a minority of studies have suggested that problematic or addictive use of SM is also associated with orthorexia nervosa risk. Future studies should prioritize examining the pathophysiology underlying these relationships to inform targeted interventions more effectively.

## Author Contributions

**Cristina Vintró‐Alcaraz:** conceptualization, data curation, investigation, writing – original draft. **Cristina Ballero Reque:** data curation, investigation, writing – original draft. **Georgios Paslakis:** resources, writing – review and editing. **Giulia Testa:** conceptualization, data curation, investigation, writing – original draft, funding acquisition.

## Conflicts of Interest

The authors declare no conflicts of interest.

## Supporting information


**Table S1**: Quality assessment of included studies.

## Data Availability

The data that support the findings of this study are available from the corresponding author upon reasonable request.

## References

[erv70027-bib-0001] American Psychiatric Association . 2022. Diagnostic and Statistical Manual of Mental Disorders. DSM‐5‐TR. 5th ed. Rev. American Psychiatric Association.

[erv70027-bib-0002] Asil, E. , M. V. Yılmaz , F. Ayyıldız , and T. Yalçın . 2023. “The Effect of Social Media Use on Orthorexia Nervosa: A Sample From Turkey.” Nutricion Hospitalaria 40, no. 2: 384–390. 10.20960/nh.04217. 36880720

[erv70027-bib-0003] Atchison, A. E. , and H. F. Zickgraf . 2022. “Orthorexia Nervosa and Eating Disorder Behaviors: A Systematic Review of the Literature.” Appetite 177: 106134. 10.1016/j.appet.2022.106134. 35750289

[erv70027-bib-0004] Awad, E. , R. Rogoza , S. Gerges , S. Obeid , and S. Hallit . 2024. “Association of Social Media Use Disorder and Orthorexia Nervosa Among Lebanese University Students: The Indirect Effect of Loneliness and Factor Structure of the Social Media Use Disorder Short Form and the Jong‐Gierveld Loneliness Scales.” Psychological Reports 127, no. 3: 1065–1084. 10.1177/00332941221132985. 36245332

[erv70027-bib-0005] Badenes‐Ribera, L. , M. Rubio‐Aparicio , J. Sánchez‐Meca , M. A. Fabris , and C. Longobardi . 2019. “The Association Between Muscle Dysmorphia and Eating Disorder Symptomatology: A Systematic Review and Meta‐Analysis.” Journal of Behavioral Addictions 8, no. 3: 351–371. 10.1556/2006.8.2019.44. 31505966 PMC7044626

[erv70027-bib-0006] Barrada, J. R. , and M. Roncero . 2018. “Bidimensional Structure of the Orthorexia: Development and Initial Validation of a New Instrument.” Anales de Psicología 34, no. 2: 282–290. 10.6018/analesps.34.2.299671.

[erv70027-bib-0007] Barthels, F. , F. Meyer , and R. Pietrowsky . 2015. “Die Düsseldorfer Orthorexie Skala–Konstruktion und Evaluation Eines Fragebogens zur Erfassung ortho‐rektischen Ernährungsverhaltens.” Zeitschrift für Klinische Psychologie und Psychotherapie 44, no. 2: 97–105. 10.1026/1616-3443/a000310.

[erv70027-bib-0008] Blanchard, L. , K. Conway‐Moore , A. Aguiar , et al. 2023. “Associations Between Social Media, Adolescent Mental Health, and Diet: A Systematic Review.” Supplement, Obesity Reviews 24, no. S2: e13631. 10.1111/obr.13631. 37753597

[erv70027-bib-0009] Bratman, S. , and D. Knight . 2000. Health Food Junkies: Overcoming the Obession With Healthful Eating. Broadway Books.

[erv70027-bib-0010] Cafri, G. , R. Olivardia , and J. K. Thompson . 2008. “Symptom Characteristics and Psychiatric Comorbidity Among Males With Muscle Dysmorphia.” Comprehensive Psychiatry 49, no. 4: 374–379. 10.1016/j.comppsych.2008.01.003. 18555058

[erv70027-bib-0011] Cataldo, I. , I. De Luca , V. Giorgetti , et al. 2021. “Fitspiration on Social Media: Body‐Image and Other Psychopathological Risks Among Young Adults. A Narrative Review.” Emerging Trends in Drugs, Addictions, and Health 1: 100010. 10.1016/j.etdah.2021.100010.

[erv70027-bib-0012] Cena, H. , F. Barthels , M. Cuzzolaro , et al. 2019. “Definition and Diagnostic Criteria for Orthorexia Nervosa: A Narrative Review of the Literature.” Eating and Weight Disorders 24, no. 2: 209–246. 10.1007/s40519-018-0606-y. 30414078

[erv70027-bib-0013] Chaki, B. , S. Pal , and A. Bandyopadhyay . 2013. “Exploring Scientific Legitimacy of Orthorexia Nervosa: A Newly Emerging Eating Disorder.” Journal of Human Sport and Exercise 8, no. 4: 1045–1053. 10.4100/jhse.2013.84.14.

[erv70027-bib-0014] Christodoulou, E. , V. Markopoulou , and A. E. Koutelidakis . 2024. “Exploring the Link Between Mindful Eating, Instagram Engagement, and Eating Disorders: A Focus on Orthorexia Nervosa.” Psychiatry International 5, no. 1: 27–38. 10.3390/psychiatryint5010003.

[erv70027-bib-0015] Cooper, M. , K. T. Eddy , J. J. Thomas , et al. 2020. “Muscle Dysmorphia: A Systematic and meta‐analytic Review of the Literature to Assess Diagnostic Validity.” International Journal of Eating Disorders 53, no. 10: 1583–1604. 10.1002/eat.23349. 32737999

[erv70027-bib-0016] Cuadrado, J. , D. Reynaud , C. Legigan , K. O’Brien , and G. Michel . 2023. “‘Muscle Pics’, a New Body‐Checking Behavior in Muscle Dysmorphia?” L’Encephale 49, no. 3: 241–247. 10.1016/j.encep.2021.11.004. 35164942

[erv70027-bib-0017] Dane, A. , and K. Bhatia . 2023. “The Social Media Diet: A Scoping Review to Investigate the Association Between Social Media, Body Image and Eating Disorders Amongst Young People.” PLOS Global Public Health 3, no. 3: e0001091. 10.1371/journal.pgph.0001091. 36962983 PMC10032524

[erv70027-bib-0018] De Oliveira, M. F. , A. Maglioni , B. A. B. Marais , L. R. Borges , L. H. M. Seraim , and A. P. Ganen . 2021. “Relationship Between Risk Behaviors for Orthorexia Nervosa, Social Media and Diets in Nutrition Students.” Saúde e Pesquisa 14: 1–14.

[erv70027-bib-0019] de Valle, M. K. , M. Gallego‐García , P. Williamson , and T. D. Wade . 2021. “Social Media, Body Image, and the Question of Causation: Meta‐Analyses of Experimental and Longitudinal Evidence.” Body Image 39: 276–292. 10.1016/j.bodyim.2021.10.001. 34695681

[erv70027-bib-0020] Dunn, T. M. , and S. Bratman . 2016. “On Orthorexia Nervosa: A Review of the Literature and Proposed Diagnostic Criteria.” Eating Behaviors 21: 11–17. 10.1016/j.eatbeh.2015.12.006. 26724459

[erv70027-bib-0021] Duradoni, M. , M. C. Gursesli , M. Fiorenza , and A. Guazzini . 2023. “The Relationship Between Orthorexia Nervosa and Obsessive Compulsive Disorder.” European Journal of Investigation in Health, Psychology and Education 13, no. 5: 861–869. 10.3390/ejihpe13050065. 37232703 PMC10216926

[erv70027-bib-0022] Eschrich, R. L. , G. Halbeisen , S. Steins‐Loeber , N. Timmesfeld , and G. Paslakis . 2025. “Investigating the Structure of Disordered Eating Symptoms in Adult Men: A Network Analysis.” European Eating Disorders Review 33, no. 1: 80–94. 10.1002/erv.3131. 39135219 PMC11617807

[erv70027-bib-0023] European Commission . 2022. The Digital Services Act. European Commission. https://commission.europa.eu/strategy‐and‐policy/priorities‐2019‐2024/europe‐fit‐digital‐age/digital‐services‐act_en.

[erv70027-bib-0024] Foster, A. C. , G. W. Shorter , and M. D. Griffiths . 2015. “Muscle Dysmorphia: Could It Be Classified as an Addiction to Body Image?” Journal of Behavioral Addictions 4, no. 1: 1–5. 10.1556/JBA.3.2014.001. PMC4394845 25592218

[erv70027-bib-0025] Ganson, K. T. , L. Hallward , R. F. Rodgers , A. Testa , D. B. Jackson , and J. M. Nagata . 2023. “Contemporary Screen Use and Symptoms of Muscle Dysmorphia Among a National Sample of Canadian Adolescents and Young Adults.” Eating and Weight Disorders 28, no. 1: 10. 10.1007/s40519-023-01550-7. 36790649 PMC9930713

[erv70027-bib-0026] Gleaves, D. , E. Graham , and S. Ambwani . 2013. “Measuring ‘Orthorexia’: Development of the Eating Habits Questionnaire.” International Journal of Educational and Psychological Assessment 12: 1–18.

[erv70027-bib-0027] Gobin, K. C. , J. S. Mills , and S. E. McComb . 2021. “The Effects of the COVID‐19 Pandemic Lockdown on Eating, Body Image, and Social Media Habits Among Women With and Without Symptoms of Orthorexia Nervosa.” Frontiers in Psychology 12: 716998. 10.3389/fpsyg.2021.716998. 34975611 PMC8714632

[erv70027-bib-0028] González, J. J. L. 2023. “Mass Media, Social Networks, and Eating Disorders: Image, Perfection, and Death.” In Eating—Pathology and Causes. IntechOpen. 10.5772/intechopen.1002270.

[erv70027-bib-0029] Griffiths, S. , and A. Stefanovski . 2019. “Thinspiration and Fitspiration in Everyday Life: An Experience Sampling Study.” Body Image 30: 135–144. 10.1016/j.bodyim.2019.07.002. 31299608

[erv70027-bib-0030] Grossbard, J. R. , C. Neighbors , and M. E. Larimer . 2011. “Perceived Norms for Thinness and Muscularity Among College Students: What Do Men and Women Really Want?” Eating Behaviors 12, no. 3: 192–199. 10.1016/j.eatbeh.2011.04.005. 21741017 PMC3134786

[erv70027-bib-0031] Hafstad, S. M. , J. Bauer , A. Harris , and S. Pallesen . 2023. “The Prevalence of Orthorexia in Exercising Populations: A Systematic Review and Meta‐Analysis.” Journal of Eating Disorders 11, no. 1: 15. 10.1186/s40337-023-00739-6. 36747235 PMC9903632

[erv70027-bib-0032] Hamurcu, T. G. Ö. , and S. Yılmaz . 2023. “The Correlation Between Orthorexia Nervosa and Social Media Use in Nursing Students.” İnönü Üniversitesi Sağlık Hizmetleri Meslek Yüksek Okulu Dergisi 11, no. 1: 1144–1158. 10.33715/inonusaglik.1156789.

[erv70027-bib-0033] Harriger, J. A. , J. A. Evans , J. K. Thompson , and T. L. Tylka . 2022. “The Dangers of the Rabbit Hole: Reflections on Social Media as a Portal Into a Distorted World of Edited Bodies and Eating Disorder Risk and the Role of Algorithms.” Body Image 41: 292–297. 10.1016/j.bodyim.2022.03.007. 35378338

[erv70027-bib-0034] Hildebrandt, T. , J. Langenbucher , and D. G. Schlundt . 2004. “Muscularity Concerns Among Men: Development of Attitudinal and Perceptual Measures.” Body Image 1, no. 2: 169–181. 10.1016/j.bodyim.2004.01.001. 18089149

[erv70027-bib-0035] Hinojo‐Lucena, F.‐J. , I. Aznar‐Díaz , M.‐P. Cáceres‐Reche , J.‐M. Trujillo‐Torres , and J.‐M. Romero‐Rodríguez . 2019. “Problematic Internet Use as a Predictor of Eating Disorders in Students: A Systematic Review and Meta‐Analysis Study.” Nutrients 11, no. 9: 2151. 10.3390/nu11092151. 31505749 PMC6769899

[erv70027-bib-0036] Imperatori, C. , A. Panno , G. A. Carbone , et al. 2022. “The Association Between Social Media Addiction and Eating Disturbances Is Mediated by Muscle Dysmorphia‐Related Symptoms: A Cross‐Sectional Study in a Sample of Young Adults.” Eating and Weight Disorders 27, no. 3: 1131–1140. 10.1007/s40519-021-01232-2. 34176075 PMC8235906

[erv70027-bib-0037] Ioannidis, K. , C. Taylor , L. Holt , et al. 2021. “Problematic Usage of the Internet and Eating Disorder and Related Psychopathology: A Multifaceted, Systematic Review and Meta‐Analysis.” Neuroscience & Biobehavioral Reviews 125: 569–581. 10.1016/j.neubiorev.2021.03.005. 33713700

[erv70027-bib-0038] Joanna Briggs Institute . 2017. Critical Appraisal Checklist for Analytical Cross Sectional Studies. Joanna Briggs Institute, JBI. https://joannabriggs.org/research/critical‐appraisal‐tools.html.

[erv70027-bib-0039] Jerónimo, F. , and E. V. Carraça . 2022. “Effects of Fitspiration Content on Body Image: A Systematic Review.” Eating and Weight Disorders 27, no. 8: 3017–3035. 10.1007/s40519-022-01505-4. 36401082 PMC9676749

[erv70027-bib-0040] Jürgensen, V. , G. Halbeisen , M. S. Lehe , and G. Paslakis . 2025. “Muscularity Concerns and Disordered Eating Symptoms in Adult Women: A Network Analysis.” European Eating Disorders Review 33, no. 5: 864–878. 10.1002/erv.3192. 40095745 PMC12319133

[erv70027-bib-0041] Kanchan, S. , and A. Gaidhane . 2023. “Social Media Role and Its Impact on Public Health: A Narrative Review.” Cureus 15, no. 1: e33737. 10.7759/cureus.33737. 36793805 PMC9925030

[erv70027-bib-0042] Karniej, P. , J. Pérez , R. Juárez‐Vela , et al. 2023. “Orthorexia Nervosa in Gay Men‐the Result of a Spanish‐Polish Eating Disorders Study.” BMC Public Health 23, no. 1: 58. 10.1186/s12889-022-14943-7. 36624429 PMC9830745

[erv70027-bib-0043] Keser, A. , A. Bayındır‐Gümüş , H. Kutlu , and E. Öztürk . 2020. “Development of the Scale of Effects of Social Media on Eating Behaviour: A Study of Validity and Reliability.” Public Health Nutrition 23, no. 10: 1677–1683. 10.1017/S1368980019004270. 32200764 PMC10200371

[erv70027-bib-0044] Levin, R. L. , J. S. Mills , S. E. McComb , and J. S. Rawana . 2023. “Examining Orthorexia Nervosa: Using Latent Profile Analysis to Explore Potential Diagnostic Classification and Subtypes in a Non‐Clinical Sample.” Appetite 181: 106398. 10.1016/j.appet.2022.106398. 36455786

[erv70027-bib-0045] Lim, M. S. C. , A. Molenaar , L. Brennan , M. Reid , and T. McCaffrey . 2022. “Young Adults’ Use of Different Social Media Platforms for Health Information: Insights From Web‐Based Conversations.” Journal of Medical Internet Research 24, no. 1: e23656. 10.2196/23656. 35040796 PMC8808344

[erv70027-bib-0046] Łucka, I. , A. Mazur , A. Łucka , I. Sarzyńska , J. Trojniak , and M. Kopańska . 2024. “Orthorexia as an Eating Disorder Spectrum—A Review of the Literature.” Nutrients 16, no. 19: 3304. 10.3390/nu16193304. 39408271 PMC11478848

[erv70027-bib-0047] Ma, L.‐L. , Y.‐Y. Wang , Z.‐H. Yang , D. Huang , H. Weng , and X.‐T. Zeng . 2020. “Methodological Quality (Risk of Bias) Assessment Tools for Primary and Secondary Medical Studies: What Are They and Which Is Better?” Military Medical Research 7, no. 1: 7. 10.1186/s40779-020-00238-8. 32111253 PMC7049186

[erv70027-bib-0048] Marks, R. J. , A. De Foe , and J. Collett . 2020. “The Pursuit of Wellness: Social Media, Body Image and Eating Disorders.” Children and Youth Services Review 119: 105659. 10.1016/j.childyouth.2020.105659.

[erv70027-bib-0049] Mazzeo, S. E. , M. Weinstock , T. N. Vashro , T. Henning , and K. Derrigo . 2024. “Mitigating Harms of Social Media for Adolescent Body Image and Eating Disorders: A Review.” Psychology Research and Behavior Management 17: 2587–2601. 10.2147/PRBM.S410600. 38978847 PMC11229793

[erv70027-bib-0050] McComb, S. E. , and J. S. Mills . 2022. “The Effect of Physical Appearance Perfectionism and Social Comparison to Thin‐Slim‐Thick‐and Fit‐Ideal Instagram Imagery on Young Women’s Body Image.” Body Image 40: 165–175. 10.1016/j.bodyim.2021.12.003. 34968854

[erv70027-bib-0051] McCreary, D. R. 2007. “The Drive for Muscularity Scale: Description, Psychometrics, and Research Findings.” In The Muscular Ideal: Psychological, Social, and Medical Perspectives, 87–106. American Psychological Association. 10.1037/11581-004.

[erv70027-bib-0052] Missbach, B. , B. Hinterbuchinger , V. Dreiseitl , S. Zellhofer , C. Kurz , and J. König . 2015. “When Eating Right, Is Measured Wrong! A Validation and Critical Examination of the ORTO‐15 Questionnaire in German.” PLoS One 10, no. 8: e0135772. 10.1371/journal.pone.0135772. 26280449 PMC4539204

[erv70027-bib-0053] Mosley, P. E. 2009. “Bigorexia: Bodybuilding and Muscle Dysmorphia.” European Eating Disorders Review 17, no. 3: 191–198. 10.1002/erv.897. 18759381

[erv70027-bib-0054] Murray, S. B. , E. Rieger , S. W. Touyz , and Y. De la Garza García Lic . 2010. “Muscle Dysmorphia and the DSM‐V Conundrum: Where Does It Belong? A Review Paper.” International Journal of Eating Disorders 43, no. 6: 483–491. 10.1002/eat.20828. 20862769

[erv70027-bib-0055] Niedzielski, A. , and N. Kaźmierczak‐Wojtaś . 2021. “Prevalence of Orthorexia Nervosa and Its Diagnostic Tools—A Literature Review.” International Journal of Environmental Research and Public Health 18, no. 10: 5488. 10.3390/ijerph18105488. 34065506 PMC8160773

[erv70027-bib-0056] Olave, L. , A. Estévez , J. Momeñe , et al. 2021. “Exercise Addiction and Muscle Dysmorphia: The Role of Emotional Dependence and Attachment.” Frontiers in Psychology 12: 681808. 10.3389/fpsyg.2021.681808. 34220650 PMC8250146

[erv70027-bib-0057] Olivardia, R. 2001. “Mirror, Mirror on the Wall, Who’s the Largest of Them All? the Features and Phenomenology of Muscle Dysmorphia.” Harvard Review of Psychiatry 9, no. 5: 254–259. 10.1080/10673220127900. 11553529

[erv70027-bib-0058] Olivardia, R. , H. G. Pope , and J. I. Hudson . 2000. “Muscle Dysmorphia in Male Weightlifters: A Case‐Control Study.” American Journal of Psychiatry 157, no. 8: 1291–1296. 10.1176/appi.ajp.157.8.1291. 10910793

[erv70027-bib-0059] Padín, P. F. , R. González‐Rodríguez , C. Verde‐Diego , and R. Vázquez‐Pérez . 2021. “Social Media and Eating Disorder Psychopathology: A Systematic Review.” Cyberpsychology: Journal of Psychosocial Research on Cyberspace 15, no. 3. 10.5817/CP2021-3-6.

[erv70027-bib-0060] Page, M. J. , J. E. McKenzie , P. M. Bossuyt , et al. 2021. “The PRISMA 2020 Statement: An Updated Guideline for Reporting Systematic Reviews.” BMJ 372: n71. 10.1136/bmj.n71. 33782057 PMC8005924

[erv70027-bib-0061] Plackett, R. , A. Blyth , and P. Schartau . 2023. “The Impact of Social Media Use Interventions on Mental Well‐Being: Systematic Review.” Journal of Medical Internet Research 25: e44922. 10.2196/44922. 37565693 PMC10457695

[erv70027-bib-0062] Pontillo, M. , V. Zanna , F. Demaria , et al. 2022. “Orthorexia Nervosa, Eating Disorders, and Obsessive‐Compulsive Disorder: A Selective Review of the Last Seven Years.” Journal of Clinical Medicine 11, no. 20: 6134. 10.3390/jcm11206134. 36294454 PMC9604819

[erv70027-bib-0063] Pope, H. G. , A. J. Gruber , P. Choi , R. Olivardia , and K. A. Phillips . 1997. “Muscle Dysmorphia. An Underrecognized Form of Body Dysmorphic Disorder.” Psychosomatics 38, no. 6: 548–557. 10.1016/S0033-3182(97)71400-2. 9427852

[erv70027-bib-0064] Pope, H. G. , D. L. Katz , and J. I. Hudson . 1993. “Anorexia Nervosa and ‘Reverse Anorexia’ Among 108 Male Bodybuilders.” Comprehensive Psychiatry 34, no. 6: 406–409. 10.1016/0010-440x(93)90066-d. 8131385

[erv70027-bib-0065] Rounds, E. G. , and L. A. Stutts . 2021. “The Impact of Fitspiration Content on Body Satisfaction and Negative Mood: An Experimental Study.” Psychology of Popular Media 10, no. 2: 267–274. 10.1037/ppm0000288.

[erv70027-bib-0066] Ryan, T. A. , T. G. Morrison , S. Roddy , and J. McCutcheon . 2011. “Psychometric Properties of the Revised Male Body Attitudes Scale Among Irish Men.” Body Image 8, no. 1: 64–69. 10.1016/j.bodyim.2010.10.004. 21095167

[erv70027-bib-0067] Şahin, C. , and M. Yağcı . 2017. “Sosyal Medya Bağimliliği ölçeği‐ Yetişkin Formu: Geçerlilik ve Güvenirlik Çalişmasi [Social Media Addiction Scale—Adult Form: Validity and Reliability Study].” Ahi Evran Üniversitesi Kırşehir Eğitim Fakültesi Dergisi 18, no. 1: 523–538.

[erv70027-bib-0068] Scheiber, R. , S. Diehl , and M. Karmasin . 2023. “Socio‐Cultural Power of Social Media on Orthorexia Nervosa: An Empirical Investigation on the Mediating Role of Thin‐Ideal and Muscular Internalization, Appearance Comparison, and Body Dissatisfaction.” Appetite 185: 106522. 10.1016/j.appet.2023.106522. 36893917

[erv70027-bib-0069] Schoenenberg, K. , and A. Martin . 2020. “Bedeutung von Instagram und Fitspiration‐Bildern für die Muskeldysmorphe Symptomatik.” Psychotherapeut 65, no. 2: 93–100. 10.1007/s00278-020-00403-3.

[erv70027-bib-0070] Schou Andreassen, C. , J. Billieux , M. D. Griffiths , et al. 2016. “The Relationship Between Addictive Use of Social Media and Video Games and Symptoms of Psychiatric Disorders: A Large‐Scale Cross‐Sectional Study.” Psychology of Addictive Behaviors: Journal of the Society of Psychologists in Addictive Behaviors 30, no. 2: 252–262. 10.1037/adb0000160. 26999354

[erv70027-bib-0071] Sener, B. S. , and H. Ozkaya . 2023. “Investigation of the Relationship Between Social Media Addiction and Orthorexia Nervosa in Adult Individuals Who Applied to Obesity Polyclinic.” ADDICTA: The Turkish Journal on Addictions 10, no. 2: 135–142.

[erv70027-bib-0072] Silva, S. C. , M. G. Elmashhara , and M. I. Sousa . 2023. “The Body Dissatisfaction Role in the Adoption of Compulsive Healthy Eating Behaviors.” International Review on Public and Nonprofit Marketing 20, no. 4: 853–873. 10.1007/s12208-022-00357-z.

[erv70027-bib-0073] Statista . 2024. Number of Social Media Users Worldwide from 2018 to 2027. Statista. https://www.statista.com/statistics/278414/number‐of‐worldwide‐social‐network‐users/.

[erv70027-bib-0074] Szczygieł, K. , and P. Podwalski . 2020. “Comorbidity of Social Media Addiction and Other Mental Disorders – An Overview.” Archives of Psychiatry and Psychotherapy 22, no. 4: 7–11. 10.12740/APP/122487.

[erv70027-bib-0075] Tarı Selçuk, K. , and C. Çevik . 2020. “Use of Dietary Supplements Among Nursing Students in Turkey in the Last 12 Months and Its Relation With Orthorexia Nervosa.” Perspectives in Psychiatric Care 56, no. 4: 885–893. 10.1111/ppc.12507. 32249454

[erv70027-bib-0076] Tarsitano, M. G. , R. Pujia , Y. Ferro , et al. 2022. “Symptoms of Orthorexia Nervosa Are Associated With Time Spent on Social Media: A Web‐Based Survey in an Italian Population Sample.” European Review for Medical and Pharmacological Sciences 26, no. 24: 9327–9335. 10.26355/eurrev_202212_30683. 36591841

[erv70027-bib-0077] Tovt, Š. , and A. Kajanová . 2021. “Introduction to Bigorexia.” KONTAKT‐Journal of Nursing & Social Sciences related to Health & Illness 23, no. 2: 133–137. 10.32725/kont.2021.014.

[erv70027-bib-0078] Turner, P. G. , and C. E. Lefevre . 2017. “Instagram Use Is Linked to Increased Symptoms of Orthorexia Nervosa.” Eating and Weight Disorders 22, no. 2: 277–284. 10.1007/s40519-017-0364-2. 28251592 PMC5440477

[erv70027-bib-0079] Tutgun‐ünal, A. , and L. Deniz . 2015. “Development of the Social Media Addiction Scale.” AJIT‐e: Academic Journal of Information Technology 6, no. 21: 51–70. 10.5824/1309-1581.2015.4.004.x.

[erv70027-bib-0080] van den Eijnden, R. J. J. M. , J. S. Lemmens , and P. M. Valkenburg . 2016. “The Social Media Disorder Scale.” Computers in Human Behavior 61: 478–487. 10.1016/j.chb.2016.03.038.

[erv70027-bib-0081] Varga, M. , B. K. Thege , S. Dukay‐Szabó , F. Túry , and E. F. van Furth . 2014. “When Eating Healthy Is Not Healthy: Orthorexia Nervosa and Its Measurement With the ORTO‐15 in Hungary.” BMC Psychiatry 14, no. 1: 59. 10.1186/1471-244X-14-59. 24581288 PMC3943279

[erv70027-bib-0082] Veritas Health Innovation . 2024. Covidence Systematic Review Software [Computer Software]. Veritas Health Innovation. https://www.covidence.org/.

[erv70027-bib-0083] Villa, M. , N. Opawsky , S. Manriquez , N. Ananías , P. Vergara‐Barra , and M. Leonario‐Rodriguez . 2022. “Orthorexia Nervosa Risk and Associated Factors Among Chilean Nutrition Students: A Pilot Study.” Journal of Eating Disorders 10, no. 1: 6. 10.1186/s40337-022-00529-6. 35016711 PMC8753887

[erv70027-bib-0084] World Health Organization . 2022. International Statistical Classification of Diseases and Related Health Problems. 11th ed. ICD‐11. World Health Organization.

[erv70027-bib-0085] Wu, Y. , E. Kemps , and I. Prichard . 2024. “Digging into Digital Buffets: A Systematic Review of eating‐related Social Media Content and Its Relationship With Body Image and Eating Behaviours.” Body Image 48: 101650. 10.1016/j.bodyim.2023.101650. 38039952

[erv70027-bib-0086] Yargic, M. P. , and M. C. Celen . 2023. “Assessing Orthorexia Nervosa by Questionnaires.” In Eating Disorders, edited by En V. B. Patel and V. R. Preedy , 1435–1449. Springer International Publishing. 10.1007/978-3-031-16691-4_84.

[erv70027-bib-0087] Yee, Z. W. , S. Griffiths , M. Fuller‐Tyszkiewicz , K. Blake , B. Richardson , and I. Krug . 2020. “The Differential Impact of Viewing Fitspiration and Thinspiration Images on Men’s Body Image Concerns: An Experimental Ecological Momentary Assessment Study.” Body Image 35: 96–107. 10.1016/j.bodyim.2020.08.008. 32977202

[erv70027-bib-0088] Yılmazel, G. 2021. “Orthorexia Tendency and Social Media Addiction Among Candidate Doctors and Nurses.” Perspectives in Psychiatric Care 57, no. 4: 1846–1852. 10.1111/ppc.12758. 33861475

[erv70027-bib-0089] Yılmazel, G. , and S. Bozdoğan . 2020. “Limited Health Literacy Increases the Risk of Orthorexia Nervosa Among Urban School Teachers.” Universa Medicina 39, no. 3: 162–170. 10.18051/univmed.2020.v39.162-170.

[erv70027-bib-0090] Yurtdaş‐Depboylu, G. , G. Kaner , and S. Özçakal . 2022. “The Association Between Social Media Addiction and Orthorexia Nervosa, Eating Attitudes, and Body Image Among Adolescents.” Eating and Weight Disorders 27, no. 8: 3725–3735. 10.1007/s40519-022-01521-4. 36562891

