# Supplementary material for: Idealized Body Images and Fitness Lifestyles on Social Media: A Systematic Review Exploring the Link Between Social Media Use and Symptoms of Orthorexia Nervosa and Muscle Dysmorphia
Source: Eur Eat Disord Rev. 2025 Aug 20;34(1):256–80. doi: 10.1002/erv.70027 (PMC12694696; doi:10.1002/erv.70027)
Supplement: Supplementary file 1 — Table S1: Quality assessment of included studies. [file ERV-34-256-s001.docx]

**Supplementary Material**

**Table S1.** Quality Assessment of Included Studies

|  | Clear research question/objective | Well-defined study population | Participation rate ≥ 50% | Subjects selected/recruited from similar populations | Sample size justification or power description provided | Exposure measured before the outcome | Sufficient timeframe for exposure-outcome association | Examined varying exposure levels | Clearly defined, valid, and reliable exposure measures | Multiple exposure assessments over time | Clearly defined, valid, and reliable outcome measures | Blinded outcome assessors | ≤ 20% loss to follow-up | Confounding variables measured and adjusted statistically | Overall Quality Rating |
| --- | --- | --- | --- | --- | --- | --- | --- | --- | --- | --- | --- | --- | --- | --- | --- |
| Asil et al., 2023 | No | Yes | Yes | Yes | Yes | No | No | Yes | Yes | No | Yes | NA | NA | No | **Fair** |
| Awad et al., 2024 | Yes | Yes | NR | Yes | Yes | No | No | Yes | Yes | No | Yes | NA | NA | Yes | **Good** |
| Christodoulou et al., 2024 | Yes | No | Yes | Yes | No | No | No | Yes | No | No | Yes | NA | NA | Yes | **Fair** |
| Cuadrado et al., 2023 | Yes | No | NR | CND | No | No | No | Yes | No | No | Yes | NA | NA | No | **Fair** |
| De Oliveira et al., 2021 | Yes | Yes | NR | Yes | No | No | No | No | No | No | Yes | NA | NA | No | **Poor** |
| Ganson et al., 2023 | Yes | Yes | NR | Yes | No | No | No | Yes | Yes | No | Yes | NA | NA | Yes | **Fair** |
| Gobin et al., 2021 | Yes | Yes | Yes | No | No | No | No | Yes | No | No | Yes | NA | NA | No | **Fair** |
|  |  |  |  |  |  |  |  |  |  |  |  |  |  |  |  |
| Hamurcu & Yılmaz, 2023 | Yes | Yes | No | Yes | Yes | No | No | Yes | Yes | No | Yes | NA | NA | No | **Fair** |
| Imperatori et al., 2022 | Yes | Yes | NR | No | Yes | No | No | Yes | Yes | No | Yes | NA | NA | Yes | **Good** |
| Karniej et al., 2023 | Yes | Yes | Yes | Yes | No | No | No | No | No | No | Yes | NA | NA | Yes | **Good** |
| Levin et al., 2023 | Yes | No | Yes | CND | No | No | No | Yes | No | No | Yes | Yes | NA | Yes | **Fair** |
|  |  |  |  |  |  |  |  |  |  |  |  |  |  |  |  |
| Scheiber et al., 2023 | Yes | Yes | Yes | Yes | No | No | No | Yes | No | No | Yes | NA | NA | NR | **Fair** |
| Schoenenberg & Martin, 2020 | Yes | Yes | CND | Yes | No | No | No | Yes | No | No | Yes | NA | NA | No | **Fair** |
| Sener & Ozkaya, 2023 | Yes | Yes | NR | Yes | No | No | No | Yes | Yes | No | Yes | NA | NA | No | **Poor** |
| Silva et al., 2023 | Yes | No | No | CND | No | No | No | CND | CND | No | Yes | NA | NA | Yes | **Poor** |
| Tarsitano et al., 2022 | Yes | Yes | Yes | Yes | Yes | No | No | Yes | CND | No | Yes | NA | NA | Yes | **Good** |
| Turner & Lefevre, 2017 | Yes | No | Yes | CND | No | No | No | Yes | No | No | Yes | NA | NA | Yes | **Fair** |
| Villa et al., 2022 | Yes | Yes | Yes | Yes | Yes | No | No | Yes | Yes | No | Yes | NA | NA | No | **Good** |
| Yee et al., 2020 | Yes | No | Yes | Yes | Yes | Yes | CND | No | No | Yes | No | No | NA | No | **Good** |
| Yılmazel & Bozdoğan, 2020 | Yes | Yes | Yes | Yes | Yes | No | No | No | No | No | Yes | NA | NA | No | **Fair** |
| Yılmazel, 2021 | Yes | Yes | Yes | Yes | Yes | No | No | Yes | Yes | No | Yes | NA | NA | No | **Good** |
| Yurtdaş-Depboylu et al., 2022 | Yes | Yes | Yes | Yes | No | No | No | Yes | Yes | No | Yes | NA | NA | Yes | **Good** |

Note. CND= cannot determine; NA=not applicable

Source for the quality assessment: National Heart Lung and Blood Institute. (2014). Quality Assessment Tool for Observational Cohort and Cross-Sectional Studies. https://www.nhlbi.nih.gov/health-pro/guidelines/in-develop/cardiovascular-risk-reduction/tools/cohort
